# Worsening functional status in nephrogeriatrics needs to be accounted for when clinically assessing CKD advancement in addition to GFR; supporting evidence based on the practical application of theoretical modelling

**DOI:** 10.1186/s12877-022-03202-4

**Published:** 2022-07-15

**Authors:** Magdalena Wisniewska, Stanislaw Niemczyk

**Affiliations:** Department of Internal Diseases, Nephrology and Dialysis Therapy, Military Institute of Medicine, Szaserow 128 Street, 04-141 Warsaw, Poland

**Keywords:** Chronic kidney disease, Nephrogeriatrics, Tinetti test, Barthel test, IADL test, Renal replacement therapy

## Abstract

The incidence of chronic kidney disease (CKD) has been found to increase with age. This has resulted in an increase in the number of elderly patients undergoing renal replacement therapy. There is a significant risk of error in making treatment decisions in patients with advanced CKD based solely on biochemical parameters of renal function, if the changes in the functional status of patients' health are not taken into account.

**Aim**

To determine the interrelated dependencies between chronic kidney disease with the functional status of patients aged over 65 years and to elucidate differences in functional status between CKD patients and controls.

**Methods**

Patient subjects were qualified according to their assessed outcomes from the study protocol, which were achieved by: geriatric interview, assessing functional status by the IADL, Barthel and Tinetti tests together with assessing kidney function by performing laboratory tests of glomerular filtration rate (GFR), creatinine and urea. Subjects were divided into two groups: method 1—according to GFR and method 2—according to GFR and functional test results. The data were statistically analysed by structural equation modelling and k-means.

**Results**

Positive relationships were found between the CKD stage and comorbidity (β = 0.55, *p* < 0.01), along with the number of medications taken and age (respectively β = 0.19, *p* = 0.001 and β = 0.30, *p* < 0.001). A highly negative relationship was observed between the CKD stage and the Tinetti test results (β = -0.71, *p* < 0.001), whilst more moderate ones were found with the IADL and Barthel scores (respectively β = -0.49, *p* < 0.001 and β = -0.40, *p* < 0.001). The patient groups demonstrated differences in health status when selected by method-2 for: age, comorbidity, number of medications taken, fitness test outcomes (Tinetti, Barthel and IADL tests at *p* < 0.005). Those groups divided according to GFR, however only showed differences in age, comorbidity and the number of medication taken (*p* < 0.005).

**Conclusions**

The functional status worsens in geriatric patients suffering from CKD. It may thus be important to also account for disruptions to functional status when assessing CKD advancement in the elderly in addition to the GFR. The biggest problems for the over 80 s suffering from CKD are gait and balance disorders, leading to a high risk of falls. Another common problem is polypharmacy, found in both the geriatric population and particularly in those suffering from CKD.

## Introduction

### Study context and background

A comprehensive assessment of health status of the elderly with CKD is not only determined by measuring markers of renal function (i.e. creatinine and GFR), but by also assessing changes in functional, emotional, cognitive and nutritional status. Such assessments require medical professionals to have the appropriate expertise and skills in order to use the appropriate scales and tests for making any geriatric assessment comprehensive, supplementing diagnoses with additional clinical information relevant for further treatment. Assessment of functional status is especially important, as undertaking renal replacement therapy in end-stage renal failure in patients with frailty syndrome may further deteriorate their functional status or shorten their survival. The poor functional state of the patient may be the reason for a decision to treat the patient with assisted peritoneal dialysis at home or to refrain from renal replacement therapy with both hemodialysis and peritoneal dialysis.

### Study aims

The study primarily aimed to answer whether CKD and functional status are related and secondly to determine what the differences are, if any, between the functional status of CKD patients compared to controls.

### A summary of relevant studies to date

Incidence rates of CKD increases with age and thus becomes a social problem in the elderly. Almost 50% of people aged over 70 years are diagnosed with CKD grade 3–5 [1]. Patients suffering from advanced CKD will be also frequently suffering from many other diseases, particularly diabetes, cardiovascular disease and vascular diseases of the central nervous system. A high morbidity rate is associated with a high mortality rate in such patients [2]. Comorbidity is mentioned by the Bansal risk prediction model, as presented in the ERBP report (European Renal Best Practice Report), where it is one of the predictors determining the probability of death in CKD patients at stages 3–5 who are not undergoing any dialysis therapy [1, 3].

Other studies have also shown that morbidity is associated with polypharmacy and mortality in the elderly [4]. Polypharmacy is taken to mean any patient taking more than five drugs, including prescription drugs, as well as the so-called dietary supplements [5]. Taking many drugs increases the risk of harmful interactions, including those directed against the kidneys which could thereby lead to acute kidney injury or an exacerbation of CKD.

Physical fitness (functional) of elderly patients is the ability to perform daily life activities, whether independently, with the aid auxiliary devices or from people such as eating and preparing meals, maintaining personal hygiene and that of their surroundings as well as moving around, the ability to take medications independently, using the telephone and exercising personal control over finances. Progressive aging however leads to a gradual loss of both fitness and being able to independently execute daily activities. A comparative study has shown that the ability of being able to carry out complex everyday activities (Instrumental Activity of Daily Living; IADL) had decreased to 9.9% in 90-year-olds compared to rates of 79.4% in those aged 65–69 years [6]. Moreover, assistance is needed by 1 out of 10 persons aged above 80 years in performing basic self-activities, such as personal hygiene and walking or eating (Activity of Daily Living; ADL); indeed, more often than not by women than men [7]. This however relates to the general population of the elderly without CKD, but there haven’t been any studies in this area on people with renal insufficiency. Mobility problems (e.g. gait and balance) contribute to falls, which affects 30–50% of the elderly living outside of care institutions, with half having falling-accidents more than once yearly [8]. The 2016 ERBP report states that CKD is associated with patients’ state of physical fitness and frailty syndrome, and therefore with the level of exercises performed and mobility [1].

### Reasons why the study is important

The presented study emphasises the role of undertaking a comprehensive geriatric assessment, particularly to detect changes in the patient's functional status when assessing CKD advancement. It also suggests a reversal from the current trend of relying only on laboratory parameters in such an assessment. This research is also highly relevant and needed because there are as yet still no guidelines for medical practitioners in geriatric nephrology on which is the most appropriate tool for assessing functional status in the elderly suffering from CKD.

## Methods

### Study protocol

The study subjects were assessed according to the developed protocol, which included the following procedures:

- 1. Geriatric interview including, *inter alia*, age, comorbidity (number of diseases), the recorded history of medications taken.
- 2. Geriatric physical examination, including:
-
  - Assessing the patient's functional status using the Lawton IADL test, the Barthel test and the Tinetti test to assess the risk of falls.
- 3. Additional blood tests for creatinine, urea and GFR assessment (short version of Modification of Diet in Renal Disease; MDRD).

### Study protocol description

Co-morbidity was evaluated by a questionnaire completed by patients during geriatric interview coupled with studying their available medical history. Questions were asked whether subjects were/had been suffering from the following medical conditions: 1) Heart attack, 2) Heart failure, 3) Hypertension, 4) Atherosclerosis, 5) CNS diseases such as stroke, TIA, 6) Hemiparesis, 7) Dementia, 8) Chronic lung disease, 9) Chronic connective tissue disease, 10) Chronic peptic ulcer disease, 11) Chronic liver disease, 12) PCHN, 13) Malignant neoplasm, 14) Chronic hematological diseases, eg. leukemia and 15) Others. One point per condition was awarded whenever confirmed by the subject.

The geriatric interview established what and how many medications were taken daily by the subjects backed up by checking the patient’s medical history notes. Only prescription medication was considered; dietary supplements were excluded. One point was awarded per medication.

The I-ADL scale was used instead of the ADL scale because this evaluates not only basic life functions, but also other complex functions as well as being more effective in those elderly patients without comorbidities. This I-ADL scale assesses eight daily activities: 1) Using the telephone, 2) Doing everyday shopping, 3) Preparing meals, 4) Daily house cleaning, 5) Loading laundry, 6) Using transport, 7) Taking one’s medication and 8) Managing one’s finances. One point was awarded per independently performed activity, with therefore a maximum score of 8 points possible. Zero points were given whenever subjects received help with these activities or whenever they hadn’t actually been engaged in. Thereby, the fewer the points, the less effectively a subject performs such everyday activities. A decrease in I-ADL scores over time indicates that a patient’s general condition is deteriorating.

The second scale used was the Barthel scale because this contains extra and important questions which are absent in the I-ADL scale concerning personal hygiene as well as ones on crucial geriatric issues such as urinary and faecal incontinence. The Barthel scale is also more suited to disabled patients. The scale evaluates ten everyday life activities as follows: 1) Eating meals, 2) Moving (from a bed to chair and back), 3) Maintaining personal hygiene (washing the face, brushing teeth, shaving), 4) Using the toilet, 5) Washing and bathing the whole thing body, 6) Walking on flat ground, 7) Walking upstairs, 8) Getting dressed and undressed, 9) Controlling urination and 10) Controlling defecation.

The following point ranges were adopted according to the scale of difficulty encountered by subjects: 5–15 points for performing activities independently, 5–10 points when assistance is given and zero points for not engaging in any given activity. The maximum possible Barthel score is thus 100 points, with 86–100 being regarded as minor, 21–85 as medium-severe and 0–20 points as a very severe patient’s condition.

The Tinetti test was used to evaluate gait and balance, as this test is regarded as being the gold standard for diagnosing disorders in such areas. A subject fully passing a balance test receives a 16 point maximum while a maximum score of 12 is awarded for the gait test; giving a total maximum score of 28 points. A score of ≤ 25 indicates that there is a risk of falls, and a score of < 19 indicates that a subject's risk of falls increases five times.

The scoring scales assessing functional status by the IADL, Barthel, Tinetti methods can be found in the footnotes at the end of this paper.

### Characteristics of the study subjects

The study group were patients aged over 65 years of age, hospitalised at the Nephrology Clinic of the Military Institute of Medicine and those patients under long-term care at the Dialysis Center, also from the Military Institute of Medicine.

According to the guidelines of KDIGO of 2012, CKD is divided according to following GFR classification:

- G1: ≥ 90 ml/min/1,73m^2 ^-normal or increased GFR.
- G2: 60–89 ml/min/1,73m^2 ^-slight reduction in GFR.
- G3a: 45–59 ml/min/1,73m^2^ -decrease in GFR between slight and moderate.
- G3b: 30–44 ml/min/1,73m^2 ^-reduction in GFR between moderate and severe.
- G4: 15–29 ml/min/1,73m^2^ -severe decrease in GFR.
- G5: < 15 ml/min/1,73m^2^ -end-stage renal disease.

This study did not adopt this division into 5 groups (according to the KDIGO guidelines) because after initial analysis of results, it turned out that patients G1 and G2 (GFR > 60 ml/min/1,73m^2^) had similar results to patients from the control group. For the purposes of this study, the division into 3 groups were thus used.

The participants were divided into groups by using two methods: 1) according to GFR division and, 2) according to division by GFR and functional test results.

The following groups were obtained when the GFR criterion was used:

- Group-1, the haemodialysis, with CKD stage V (GFR of <15 ml/min/1.73m^2^); *n*=28 (where n is number of participants of the group).
- Group-2, with CKD stage III-IV (GFR of 15-59 ml/min/1.73m^2^); n=62.
- Group-3, controls, subjects without CKD, n=23, patients with a GFR of ≥60ml /min/1.73m^2^ were qualified for this study, without any past history of CKD nor with any additional tests suggesting a past CKD diagnosis).

The following groups were obtained when the GFR criterion was used together with functional test outcomes:

- Group i.1 with CKD stage IV-V (GFR of 0-29 ml/min/1,73m^2^), n=28, who were on haemodialysis together with n=16 subjects with CKD stage IV that had similar functional test results; giving a total of n=44.

- Group i.2 with CKD stage II-III (GFR of 30-49 ml/min/1,73m^2^ ) and similar functional test results; *n*= 34.

- Group i.3, the controls, *n*=23, CKD-absent and *n*=12 subjects with a GFR of 50-59 ml/min/1,73m^2^ that had similar functional test outcomes to those with CKD absent; giving a total of *n*=35.

The group breakdowns distinguished 3 clusters of patients, corresponding to the 3 patient groups so formed by the primary division according to the GFR described earlier: the CKD group, the hemodialysis group and the control group. Similar results were observed for each group in terms of both renal function markers and the geriatric evaluation tests.

Please note that the MDRD formula was used to calculate the GFR because it is the most common and used one every day in our center. Of course, formulas using Cystatin C are recommended for calculating GFR in the elderly, but their use is not as accessible as MDRD.

### Statistics methods

Structural equation modeling was used to statistically measure and analyze the theoretical model adopted, which allows the causal relationships of observed and the underlying latent variables to be flexibly and conveniently mapped according to theoretical reasoning behind such relationships. This method is based on assessing the fit of the empirical variance–covariance matrix, (from the observed data) to the theoretical matrix (designed by the researcher). The stronger the fit, the more closely the data matches the researcher's reasoning as expressed in the structure of the theoretical model. The structural model itself was estimated by the IBM AMOS v.13 using the asymptotically distribution free (ADF) method [9]. The k-means grouping method was used to group patients according to the variables used for building the structural model. This grouping method is based on analyzing how close the Euclid distances are of given observations to the calculated cluster centers; the smaller such distances the more strongly are observations classified to the cluster [10]. The algorithm used aims to minimize the variance within clusters and to maximize the variance between clusters. From the medical point of view, 3 groups of patients were envisaged, therefore, in the k-means analysis, a 3-cluster analysis was forced through. Statistica v.13 software was used for this analysis.

Selected multi-group comparisons of significance were performed by ANOVA (analysis of variance) which determines whether there are any statistically significant differences between independent groups of quantitative variables. It compares intra-group variation with inter-group variation [11]. It found significant, post-hoc testing is used to locate which differences are then significant. This study used the LSD (least significant difference) test for this purpose, to verify the significance of differences between groups per analysed variable.

## Results

### Theoretical model

**Fig. 1:**
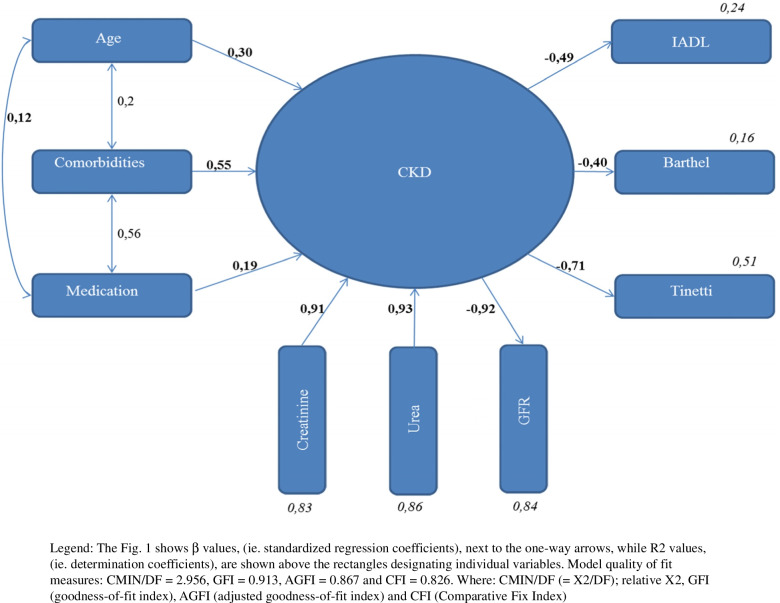
The theoretical model in the author's own elaboration describing the interrelationships between CKD and the patient's age, comorbidity, the number of medications taken and the functional status measured using the IADL, Tinetti, and Barthel tests

The theoretical model chosen for this study was based on scientific studies that shows the relationship of CKD with patient age, comorbidity, number of medications taken and functional status. The patient's age, comorbidity and the number of medications taken are considered exogenous variables (i.e. external, independent) in the model of chronic kidney disease. We assumed that when these factors rise, their increase was positively related to the CKD stage (i.e. a positive relationship). However, chronic kidney disease is also a variable in the theoretical model concerning the patient's physical condition; understood as being physical fitness. The theoretical model assumes a negative relationship between CKD and functional status, i.e. the higher the CKD stage, the worse functional status; i.e. a negative relationship. The analysis using structural equations revealed a weak relationship between CKD and the number of medications (β = 0.19, *p* < 0.01), demonstrating that medications taken accounted for 3.8% of the variability in CKD scores (R^2^ = 0.03). A moderate relationship between comorbidity and CKD was found at β = 0.55, *p* < 0.001, showing that comorbidity is accounted for by 30% of the variability in the CKD scores (R^2^ = 0.30). The relationship between age and CKD turned out to be weak at β = 0.30, *p* < 0.001, meaning that patient age accounts for 9% of the variability in CKD scores (R^2^ = 0.09). The number of medications taken and comorbidity were found to be moderately related to each other at β = 0.56, *p* < 0.001, but there was no relationship observed between the number of medications taken and age, nor was there one between comorbidity and age; both being statistically insignificant. CKD is negatively associated on how patients cope with daily activities as measured by the I-ADL test, (β = -0.49, *p* < 0.001), which shows that CKD accounts for 24% of the variability in patient scores (R^2^ = 0,24), meaning that the higher the CKD stage, the worse patients are able to cope with everyday activities. CKD is also negatively associated with how patients cope with daily activities as measured by the Barthel test. This relationship turned out to be moderate (β = -0.40, *p* < 0.001), with CKD accounting for 16% of the variability by this test (R^2^ = 0,16). The higher the CKD stage, the worse the patient is able to cope with everyday activities. In addition, CKD is negatively related to the patient's gait and balance, where CKD is strongly associated with the Tinetti test values, (β = -0.71, *p* < 0.001), indicating that CKD explains 51.2% of the variability in patients' scores in this test (R^2^ = 0,51). Thus the higher the CKD stage, the greater gait and balance are impaired. The described results are presented in Fig. 1 and Table 1.

**Table 1:** The results of the theoretical model describing the interrelationships between CKD and the patient's age, comorbidity, the number of medications taken and the functional status measured using the IADL, Tinetti, and Barthel tests

Dependent variable	Impact direction	Independent variable	β	B	S.E	Z	P	R^2^
CKD	< –-	Comorbidities	,55	,33	,05	6,66	***	0,30
CKD	< –-	Age	,30	,06	,01	5,41	***	0,09
CKD	< –-	Medication	,19	,093	,03	2,68	***	0,03
GFR	< –-	CKD	-,91	-20,47	1,01	-20,14	***	0,83
Urea	< –-	CKD	,92	29,65	1,47	20,09	***	0,86
Creatinine	< –-	CKD	,91	1,00			***	0,82
IADL	< –-	CKD	-,49	-,48	,060	-8,11	***	0,24
Barthel	< –-	CKD	-,40	-3,14	,47	-6,65	***	0,16
Tinetti	< –-	CKD	-,71	-2,96	,20	-14,49	***	0,51

β - Beta standarised coefficient, B - Beta unstandarised coefficient (in units of measurements), *S.E.-* Standard error of B, *Z* - Value of Z distribution statistic, *p -* significance

R.^2^ - coefficient of determination

^***^ *p* < 0.001

^**^ *p* < 0.01

^*^ *p* < 0.05

### Group results

#### Group outcomes divided according to GFR and other variables

**Fig. 2:**
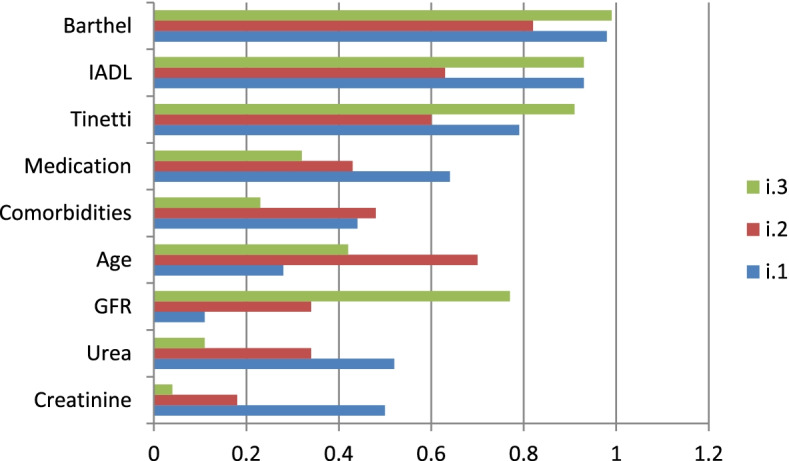
Normalized means for individual variables in groups divided according to GFR and other variables. Legend: i.1 group – blue colour, i.2 group-red colour, i.3 group –green colour. X-axis: Change to decimal point notation, ie. 1.2, 1.0, 0.8, 0.6, 0.4, 0.2 & 0.0. Y-axis: Creatinine, Urea, GFR, Age, Comorbidities, Medication, Tinetti, IADL, Barthel

Three similar clusters of test subjects were distinguished according to the intensity of variables as described in the theoretical model. The first group (i.1) consisted of 44 subjects who had the highest concentrations of creatinine (normalised mean value) and urea but the lowest GFR i.e. Those with advanced CKD stage IV-V. This group had the lowest mean age among all the groups distinguished but the highest comorbidity and they took the most drugs. Outcomes from the Tinetti test were average, whilst they were high for everyday activities (according to the IADL and Barthel tests) (Fig. 2, Table 2).

**Table 2:** Cluster group arithmetic mean values according to GFR and function tests

Variables	Units	Groups		
		**i.1**	**i.2**	**i.3**
Creatinine	mg/dl	4.98	2.13	1.07
Urea	mg/dl	129.45	92.11	43.49
GFR	ml/min/1,73m^2^	13.43	34.39	69.77
Age	years	72.43	84.64	76.8
Comorbidities	number of diseases	5.22	4.55	2.8
Medication	number of drugs	10.15	7.02	5.42
Tinetti	points	22.65	17.38	26.6
IADL	points	7.54	5.5	7,6
Barthel	points	97.5	82.05	99.14
Number of cases	Number = *n*; (percantage)	44 (38.93)	34 (30.08)	35 (30.97)
Sum of all cases	Number	113		

A second cluster was classified by algorithms into 34 people with average study levels of creatinine, urea and GFR; this being subjects with stage II-III CKD (Fig. 2,Table 2). This group (i.2) had the highest mean study age, the highest score comorbidity ascores of all groups, medication at average study levels and had the lowest Tinetti test scores as well as on the daily activities tests (IADL, Barthel).

The algorithms classified 35 subjects in the third cluster with the lowest creatinine and urea concentrations but the highest GFR value. These were subjects without CKD (group i.3, Fig. 2, Table 2). They had average ages compared to the other groups, the lowest comorbidity and took the least drugs, achieving the highest outcomes in the Tinetti test as well as the highest scoring of daily activities by the IADL and Barthel tests.

These clusters so identified showed statistically significant differences with in all variables (Table 3). Large effects between the clusters were observed for creatinine, urea, GFR, age, comorbidity, medications and the Tinetti, IADL and Barthel test scores.There were significantly differences in all variables between the identified;

**Table 3:** ANOVA performed on variables between groups divided according to GFR and function testing

	Units	Intra-group variation	f	Inter- group variation	df	F	p-value	η^2^	Int
Creatinine	mg/dl	329.1	2	188.6	10	95.9	< 0.005	0.635	Large
Urea	mg/dl	144,064.9	2	122,889.8	10	64.4	< 0.005	0.539	Large
GFR	ml/min/1,73m^2^	62,245.8	2	23,286	10	147	< 0.005	0.727	Large
Age	years	2883.9	2	3752.2	10	42.2	< 0.005	0.434	Large
Comorbidities	number of diseases	118.8	2	469.7	10	13.9	< 0.005	0.201	Large
Medication	number of drugs	461.6	2	909.4	10	27.9	< 0.005	0.336	Large
Tinetti	points	1475	2	3252.3	10	24.9	< 0.005	0.312	Large
IADL	points	101.9	2	241.8	10	23.1	< 0.005	0.296	Large
Barthel	points	6267	2	33,505.2	10	10.2	< 0.005	0.157	Large

Description of symbols used in Tables 3 & 5 (and in text): df = degrees of freedom (either between or within groups), F = variance ratio, η^2^ = a measure of the proportion of total variance (related to the effect size index), Int = the magnitude of the effect size index, i.e.; 0.01–0.06 (Small), 0.06–0.14 (Medium) and > 0.14 (Large), [12].

#### Group outcomes divided according to GFR

A cluster analysis was also performed on the variables described in the theoretical model by using the k-means method where a modelled grouping of variables was introduced for those undergoing dialysis, with and without renal failure, in such a way that only subjects belonging to groups selected on the basis of GFR values were classified to the clusters.

**Fig. 3:**
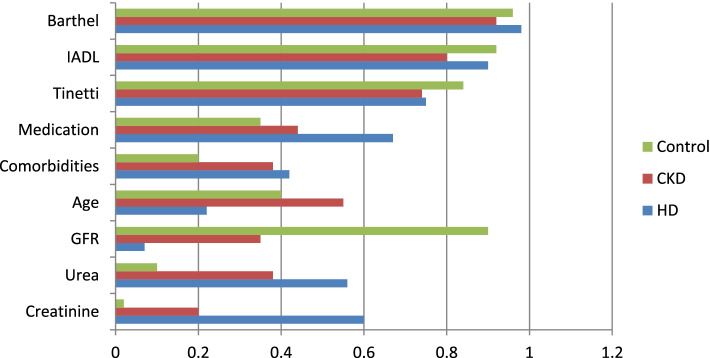
Grouped clusters developed by the k-means method according to GFR. Legend: HD (group 1), CKD (group 2), Control (group 3). X-axis: Change to decimal point notation, i.e. 1.2, 1.0, 0.8, 0.6, 0.4, 0.2 & 0.0. Y-axis: Creatinine, Urea, GFR, Age, Comorbidities, Medication, Tinetti, IADL, Barthel

This aimed to determine whether there are significant differences between these groups and what profile is characteristic for each of these groups, in terms of the variables described in the theoretical model. The solution to the cluster analysis is presented in Fig. 3.

People belonging to the group with renal insufficiency (group2, *n* = 62, Table 4) have average results among all groups in terms of creatinine, urea and GFR, they are characterized by the highest mean age, lower than the group of patients on hemodialysis (group 1, *n* = 28, Table 4) comorbidity and the number of drugs taken. The remaining differences between the groups were statistically insignificant (Table 5).

**Table 4:** Cluster means in groups according to GFR

Variables	Units	Groups		
		**1**	**2**	**3**
Creatinine	mg/dl	5.87	2.31	0.92
Urea	mg/dl	130.35	93.98	38
GFR	ml/min/1,73m^2^	10.35	32.62	82.2
Age	years	71.71	80.4	76.5
Comorbidities	number of diseases	5.28	4.45	2.56
Medication	number of drugs	10.78	7.19	5.56
Tinetti	points	21.89	21.66	24.5
IADL	points	7.25	6.66	7.34
Barthel	points	96.78	91.53	94.1
Number of cases	Number = *n*; (percantage)	28(24,77)	62(54,86)	23(20,35)
Sum of all cases	Number	113

**Table 5:** ANOVA performed on variables between groups divided according to GFR

	Units	Inter-group variation	df	Intra-group variation	D f	F	p-value	η^2^	Int
Creatinine	mg/dl	358.8	2	158.9	110	124.19	< 0.005	0.69	Large
Urea	mg/dl	108,575.5	2	158,379.2	110	37.7	< 0.005	0.4	Large
GFR	ml/min/1,73m^2^	67,994.3	2	17,537.5	110	213.23	< 0.005	0.79	Large
Age	years	1481.7	2	51,544	110	15.81	< 0.005	0.22	Large
Comorbidities	number of diseases	97.8	2	490.7	110	10.95	< 0.005	0.16	Large
Medication	number of drugs	387	2	984	110	21.63	< 0.005	0.28	Large
Tinetti	points	139.1	2	4588.3	110	1.66	0.19	0.02	Small
IADL	points	11.3	2	332.4	110	1.87	0.16	0.03	Small
Barthel	points	549.4	2	39,222.8	110	0.77	0.47	0.01	Small

Control subjects, without CKD (group 3, n = 23, Table 4), had the lowest creatinine and urea, whilst having the highest GFR. They were of average study age, had the lowest comorbidity index and took the fewest drugs. All other differences found between the groups were statistically insignificant (Table 5).

The differences between groups were large for creatinine, urea, GFR, age, comorbidity, and medications, but small for Tinetti test outcomes and those measuring daily activities (i.e. I-ADL and Barthel tests) (Table 5).

Description of symbols used in Tables 3 & 5 (and in text): df = degrees of freedom (either between or within groups), F = variance ratio, η^2^ = a measure of the proportion of total variance (related to the effect size index), Int. = the magnitude of the effect size index, i.e.; 0.01–0.06 (Small), 0.06–0.14 (Medium) and > 0.14 (Large), [12].

## Discussion

### Relationship of chronic kidney disease with functional status

The analysis using structural equations demonstrated the different extents by which CKD is associated with all the studied variables in the elderly (i.e. numbers of medications taken, comorbidity, age and physical fitness).

As an independent factor, having CKD worsens the physical condition of elderly patients and results in a two-fold increase in the risk of impaired functional status compared to those elderly people without CKD (13–17).

The mechanisms underlying this relationship are complex. Some studies have associated CKD with increased comorbidity, especially with cardiovascular diseases, which in turn reduces physical activity and thereby an impaired functional status (18). Indeed, our study showed that CKD is moderately associated with comorbidity and patient performance in functional tests. Other studies have explained this association by the presence of the so-called fragility syndrome which occurs three times more often in patients with CKD and end-stage renal disease than subjects with normal renal function (14, 19, 20). The fragility phenotype is characterised by a tendency for unintentional weight loss, muscle weakness, subjective exhaustion, slow walking speed and decreased physical activity (21). One of its causes is considered to be high comorbidity. It is also responsible for increasing the body’s energy consumption thereby leading calorific deficiency and, consequently to protein-energy malnutrition. Other causes of the fragility syndrome in CKD include anaemia, chronic inflammation, metabolic acidosis, endocrine and calcium-phosphate disorders (22). Remedying such disorders improves the functional status in post-kidney transplant patients and is another piece of evidence to show that CKD worsens the functional status of patients (23).

The relationship between CKD and patient age is unclear. A multicentre meta-analysis by S. Hallan (JAMA in 2012), demonstrated that low GFR and high albuminuria are associated with mortality and ESRD (End-Stage Renal Disease) irrespective of age (24). Nonetheless, our study in fact showed an association of CKD with patient age. Reasons for this disagreement are most likely because mean age in the former study was 49.4 years compared to our study mean of 80.4 years.

The problem of polypharmacy is common among older patients, but there are only a few scientific reports regarding any direct relationship between the number of medications taken with CKD. The presented study however showed a weak relationship between CKD and the number of medications taken, whereas an English study showed that approximately 20% of patients with CKD take more than 10 different medications daily (25).

### Differences in patients’ health status in groups divided according to GFR and functional test outcomes (groups i.1, i.2, i.3)

Our study has revealed that the patient groups i.1, i.2, i.3 significantly differed from each other in terms of: age, comorbidity, number of medications taken and fitness test outcomes (Tinetti test, Barthel's scale, IADL scale); *p* < 0.005. As expected, i.3 group outcomes showed the lowest comorbidity, patients taking the least drugs, highest scoring in gait and balance tests (Tinetti test) along with the fitness tests (IADL, Barthel).

Patients from i.1 group are however younger, have the highest comorbidity, take the most medications, and have average study results in for the fitness tests. The average study outcomes in tests of daily activities in this group can be explained by their lower age compared to the ages of the i.2 subjects (72.4 years vs 84 years). Nevertheless, the high scores from the IADL test (7.5 pt/ 8pt.) and Barthel test (97.5 pt/ 100pt) did not correlate with the low score in the Tinnetti test (22.6pt /28 pt). These results suggest there is an additional factor present that increases the risk of falls within this patient group; this might be the large number of drugs taken (average of 10 /day) or the high concentrations of creatinine (average 4.98 mg /dl) or urea (average 129.45 mg /dl).

The i.2 group have the highest mean age (84 years), take less drugs than dialysis patients, and have a lower comorbidity compared to dialysis patients. Furthermore, they scored the lowest in the gait and balance test (17.3pt / 28pt in the Tinetti test) and also the lowest results in the fitness tests (IADL test, Barthel). Such low scoring in the functional tests are due to the presence of advanced CKD (stage III) and the age of the respondents. Similar observations have been presented by D. Weiner and S. Seliger (26).

One can assume that the low scores obtained by patients aged over 80 years in the Tinetti test will lead to more frequent falls in these patients. Indeed, yearly falls are observed in around 30% of people aged over 75 years without CKD, in about 38% of people with CKD and about 50% of patients on haemodialysis (27–29). Disorders in gait arise from the following in these patients: weakening of muscle strength when suffering from the fragility syndrome, sensation impairment during diabetic neuropathy, visual disturbances whilst suffering from cataracts, glaucoma, macular degeneration, diabetic and hypertensive retinopathy, disorders of the vestibular system (caused by hyper- and arterial hypotension), water and electrolyte disruption, polypharmacy and cerebral circulatory disorders.

Both CKD patient groups, (i.e. stage III of CKD and the HD—stage IV-V of CKD) gave more inferior test scores for the functional test compared to controls thus providing evidence that CKD significantly worsens the functional status of patients irrespective of age.

### Differences in patients’ groups health status divided according to GFR (groups 1,2,3)

Our results demonstrate that those subject groups with renal failure, without renal failure and those undergoing haemodialysis significantly differ from each other in terms of: age, comorbidity and number of medications taken; *p* < 0.005. Differences in daily activities were found to be insignificant as measured by the Tinetti, IADL and Barthel scales which shows that assessing of the health of patients with CKD should be made not only on the basis of GFR values, but also by assessing changes in the functional status of patients.

Finally, the amount of medications taken by the patient groups also needs commenting on. The most drugs were taken by the HD patients (~ 10), regardless of group breakdown, whilst slightly fewer were taken by CKD patients ( approx. 7), and the least by the control group (approx. 5). This means that the problem of polypharmacy concerned all subjects and therefore it is necessary to take remedial measures to reduce this unfavourable phenomenon.

## Conclusions

CKD in the geriatric population worsens their functional status. The causal relationship, (ie. linking the functional state with the advancement of CKD), has been confirmed and proven by both the results using the theoretical model and the results of the cluster analysis. This indicates that the more CKD is advanced, then the worse the functional state is. Furthermore, subjects aged over 80 years with CKD (being at least in stage III), achieved the worst results in the gait and balance disorders test (Tinetti test). It is in fact these disorders that particularly need to monitored when assessing the functional status of such patients. Polypharmacy is also another common problem in the elderly, especially in patients with CKD.

## Data, materials used

https://doi.org/10.1111/j.1532-5415.1986.tb05480.x

https://www.leadingagemn.org/assets/docs/Tinetti-Balance-Gait--POMA.pdf

https://doi.org/10.1093/geront/9.3_Part_1.179

https://www.alz.org/careplanning/downloads/lawton-iadl.pdf

http://www.sciepub.com/reference/163871

https://stopstroke.massgeneral.org/pdfs/barthel_reprint.pdf

## Data Availability

The raw data of the current study are not publicly available due to the protection of the participants personal information but are available from the corresponding author on reasonable request.

## Abbreviations

CKD: Chronic Kidney Disease
GFR: Glomerular Filtration Rate
IADL: Instrumental Activity of Daily Living
ADL: Activity of Daily Living
ERBP: European Renal Best Practice
MDRD: Modification of Diet in Renal Disease
